# A practical and safer model of nitrogen mustard injury in cornea

**DOI:** 10.1371/journal.pone.0327622

**Published:** 2025-07-03

**Authors:** Ana M. Sandoval-Castellanos, Yao Ke, Tiffany M. Dam, Emanual Michael Maverakis, Mark J. Mannis, Xiao-Jing Wang, Min Zhao

**Affiliations:** 1 Department of Ophthalmology & Vision Science, School of Medicine, University of California, Davis, California, United States of America; 2 Department of Pathology and Laboratory Medicine, School of Medicine, University of California, Davis, California, United States of America; 3 VA Northern California Health Care System, Mather, California, United States of America; 4 Department of Dermatology, Institute for Regenerative Cures, School of Medicine, University of California, Davis, California, United States of America; University of Missouri-Columbia, UNITED STATES OF AMERICA

## Abstract

**Purpose:**

Sulfur mustard (SM) is an alkylating agent used in warfare and terrorism that inflicts devastating ocular injuries. Although the clinical symptoms are well described, the underlying mechanisms are not fully understood, hindering the development of effective treatments. One major roadblock is the lack of a suitable model due to the extremely hazardous nature of SM, which requires strict safety measures. As a safer and practical alternative, we report a novel model that uses mechlorethamine (nitrogen mustard) gel, an FDA-approved topical chemotherapeutic administered by patients at home. Here we demonstrate its suitability to induce mustard corneal injury in any laboratory.

**Methods:**

*Ex vivo* porcine corneas were injured with mechlorethamine gel. Hematoxylin-eosin staining and immunohistochemistry were performed to evaluate histopathology of SM-like corneal injuries: epithelium thickness and stromal separation, keratocyte and inflammatory cell counts, and expression of inflammation and fibrosis markers.

**Results:**

This model showed the characteristic histopathology and expression of cyclooxygenase-2 (inflammation) and fibronectin-1 (fibrosis), which were consistent with other well-established SM-like corneal injury models.

**Conclusion:**

Given its ease of implementation and safety, this mechlorethamine model could be used to study the full course of mustard corneal injuries. This model is expected to facilitate the understanding of mustard ocular injuries and the development of novel therapeutics.

**Translational relevance:**

This model will allow safe evaluation of SM-like corneal injuries within 24 hours, facilitating the identification of early/new molecules that might help to develop novel treatments.

## 1. Introduction

Sulfur mustard (bis[2-chloroethyl]sulfide; SM) is an alkylating agent used in warfare and terrorism, which has inflicted thousands of devastating ocular injuries, affecting the quality of people’s lives [1,2]. SM was first used in WWI at Ypres (1917), and most recently in the Iran-Iraq conflict (1980–1988) and the Syrian Civil War (2011-present) [2–5]. SM is a low-cost and accessible chemical agent, easily synthesized and often stockpiled [2]. Disseminated as vapor or liquid droplets, SM persists as a threat to soldiers and civilians around the world [2]. The severity of the SM ocular injury varies, depending on dosage and exposure time [6,7].

Clinically, SM ocular injuries, known as mustard gas keratopathy (MGK), manifest in two phases: an acute phase with symptoms such as eye pain, photophobia, decreased vision, conjunctivitis, and lacrimation; and a chronic phase, where these symptoms reappear alongside new ones, such as neovascularization, edema, corneal opacification, ulceration, dry eye, limbal stem cell deficiency, and even blindness) [8–14]. Current medical treatments include daily irrigation, pain management, anti-inflammatories, antibiotics, limbal stem cell transplantation, and amniotic membrane transplantation, but there is no effective cure for MGK, and hence, suitable therapies are yet to be developed [2,5,11,13].

The most accepted theory of action is that SM alkylates DNA, RNA, proteins, and lipid membranes. SM undergoes cyclization and forms ethylene sulfonium, which is later converted to carbonium ions that react with DNA, RNA, and proteins. Cells try to repair DNA by activating poly (ADP-ribose) polymerase (PARP). However, excessive PARP activity causes a reduction of nicotinamide adenine dinucleotide (NAD^+^), decreasing glycolysis. This process hinders energy production, causing cell death [11,15–17]. Even though angiogenesis, fibrosis, oxidative stress, inflammation (through the production of cyclooxygenase-1 (COX-1) and COX-2)), and expression of fibronectin and matrix metalloproteinases (MMPs) are indicators of corneal SM injury, the biological mechanisms responsible for MGK are not fully understood [7,8,10,12–14].

Aside from the complexity of the mechanism of action of SM, another roadblock arises while studying SM-induced injuries: SM is an extremely hazardous material, which requires highly controlled research environments and strict regulations and permits that are not available to most ocular research laboratories in the USA [18,19]. SM presents a grave danger to scientists, as accidental exposure can be catastrophic [5]. For this reason, biologically relevant models to investigate the mechanistic effects of SM are scarce [13]. Nitrogen mustard (bis(2-chloroethyl) methylamine, NM) is also an alkylating agent, analogous to SM [13,20]. NM, like SM, modifies DNA, proteins, and other molecules, causing ocular injuries similar to exposure to SM [13]. Despite NM being commercially available, it has the disadvantage that it is a very toxic agent that causes corrosion, acute dermal and ocular toxicity, and possesses severe mutagenic and carcinogenic properties [21]. In addition, the NM reagent is sold as a powder; hence, preparation is needed, increasing the risk of eye, skin, and pulmonary exposure. Therefore, it is imperative to find an agent that can mimic the disastrous effects of SM or NM, without hindering the researchers’ health and safety.

Current mustard injury models, both *ex vivo* and *in vivo*, use vapor SM or liquid NM and study the pathology, histopathology, and molecular expression of diverse biological pathway mechanisms. The animals used in these models are mice, rats, bovines, and rabbits. However, even though corneas from rats and mice and their specific reagents are available, their anatomy differs from human corneas [9,12,22]. The use of rabbit eyes has proven to be advantageous due to their anatomical similarities to the human eye. Nevertheless, rabbit corneas are more resistant to SM injury due mainly to differences in the cornea’s permeability [12,23]. Porcine corneas are emerging as a corneal tissue of choice because they are biologically similar to human corneas, cost-effective, readily available, easy to handle, and follow the 3Rs principle in animal research (replacement, reduction, and refinement) [24–26].

Our scientific question was whether we could develop an NM-induced cornea injury model using a safer alternative to SM and liquid NM. Therefore, we developed a model using 0.016% mechlorethamine gel, also known as NM. Mechlorethamine gel is currently used for the topical treatment of stage IA and IB mycosis fungoid-type cutaneous T-cell lymphoma [27]. Mechlorethamine gel is safe for patients to use in their homes [28–30]; hence, it is a safe drug to handle in the laboratory without the need for specialized protective equipment or approved facilities.

Herein, we report a safer, novel, and practical NM-induced corneal injury model. We delivered an NM injury, using topical mechlorethamine on *ex vivo* porcine corneas. We observed that changes in epithelial thickness, loss of epithelial layer (de-epithelialization), epithelial-stroma separation, and decreased keratocyte cell count were present in the corneal tissue. To further validate this model, we evaluated the production of COX-2 and fibronectin 1 (FN1) by immunohistochemistry (IHC), as SM and NM injuries induce inflammation and fibrosis. Our results show an increased expression of both markers after NM exposure.

## 2. Materials and methods

### 2.1. Corneal tissue

All studies here were done in accordance with the University of California Davis’s Institutional Animal Care and Use Committee (IACUC), which states that an approved protocol was not needed as the experiments were performed only with animal tissue obtained from slaughterhouses.

**Fig 1:**
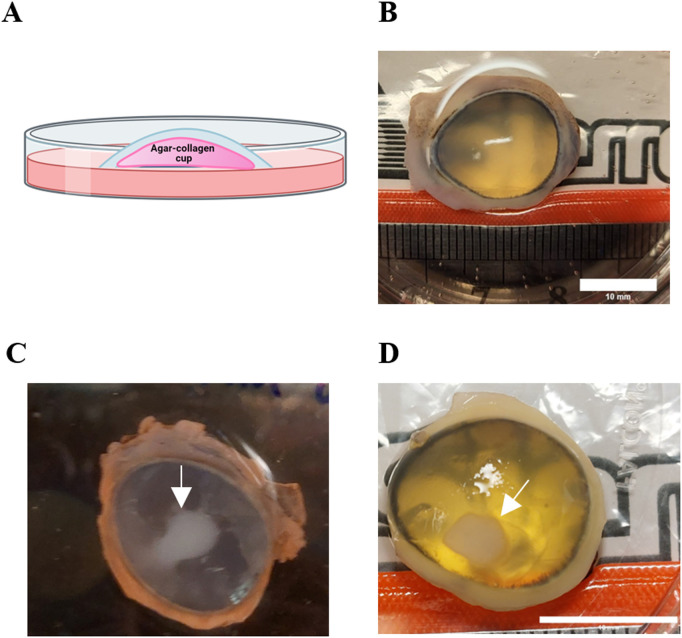
Mechlorethamine gel exposure induces de-epithelialization and opacity of the cornea, typically seen in nitrogen mustard (NM)-induced corneal injury. A. Schematic diagram showing the organ culture of the porcine cornea (blue) at the air-liquid interface. B. Organ culture image of a healthy, clear, uninjured porcine cornea, viewed from above. C. and D. A porcine cornea with an NM injury. An opaque area is seen at the site of injury (white arrow). The image was taken immediately after being wounded. Scale bars = 10 mm.

Porcine eyes (from male and female pigs, 180−300 lbs.) were purchased from Sierra Medical Inc. (Whittier, CA, USA) and the Meat Laboratory at UC Davis (Department of Animal Science, Davis, CA, USA). Eyes were processed immediately after arrival. Excess tissue (fat, muscle, connective tissue) was removed using surgical scissors. Then, globes were rinsed twice with sterile phosphate-buffered saline (PBS, Amresco, Cat No. E404-200TABS, USA). For excising the corneas, the protocol by Castro et al. [31] was followed, with some modifications: the globe was held with a tissue (Kimwipes, Kimberly-Clark Professional, USA), and the cornea was excised from the eyeball using a no. 11 blade by making an incision and cutting ~ 2 mm from the edge of the cornea, to include the limbus. The cornea was then placed upside down in a Petri dish with PBS. Then, with two pairs of forceps, the cornea was held upside down, forming a cup, and filled with warmed 1% (w/v) agar (Sigma-Aldrich, Cat No. A6686, Germany) with 1 mg/mL collagen (PureCol Type I collagen solution (bovine), Advance Biomatrix, Cat No. 5005, USA) solution in DMEM/F12 (Dulbecco’s Modified Eagle Medium/Ham’s F-12, Life Technologies, Cat No. 11330032, USA) to maintain the corneal curvature. When the agar-collagen solution hardened, the cornea was placed right side up in a Petri dish and culture medium was added until the limbus was covered, creating an air-liquid interface (see Fig 1A). Corneas were cultured at 37°C with 5% CO_2_ for a recovery period of 24 h. See Table in S1 Table for culture medium composition.

### 2.2. Injuring the cornea with mechlorethamine gel (NM)

Corneas were allowed to recover and stabilize for 24 h before inducing NM injury. Mechlorethamine 0.016% gel (brand name Valchlor^®^, Helsinn Therapeutics, USA) was applied to the corneas as follows: ~ 8 mg of 0.016% mechlorethamine gel was added to a 3 mm filter paper disk (Whatman^®^, USA), placed on the cornea, and incubated for 5 or 15 minutes at 37°C/ 5% CO_2_. Controls were: i) unwounded corneas with no treatment; ii) corneas treated with filter paper only (FP). Then, corneas were rinsed three times with PBS and fixed immediately after wounding with 10% (w/v) paraformaldehyde (PFA, Sigma-Aldrich, Cat No. P6148, Germany) and 1% (v/v) glutaraldehyde (Sigma-Aldrich, USA) solution in PBS.

### 2.3. Histology

After fixing, corneas were paraffin-embedded and sectioned into 10 µm sections, then mounted on glass slides. Sections were stained with hematoxylin and eosin (H&E). Images of the cross-sections were taken using an Olympus microscope (Olympus BX43) and cellSens Dimension software (Olympus). Images were taken at magnifications of 4x, 10x, and 20x to observe any structural changes in epithelial thickness, epithelial loss, epithelial-stroma separation, keratocyte cells, and inflammation cell count as a consequence of mechlorethamine exposure. At least 3 corneas were imaged per condition. ImageJ (version 1.53e, National Institutes of Health, USA) was used for measurements.

Epithelium thickness was determined by calculating the average thickness of at least five separate measurements in the wounded area per sample. The percentage of epithelium-stroma separation was calculated as = (length of total epithelial separation ÷ entire cornea length) × 100.

For stroma cell count, we adapted the methodology used by Goswami et al. [6]: the total number of cells in the stroma was estimated from 3 different stromal areas (1 mm^2^ each), in the injury site of each cornea. A cell in the stroma with a flat nucleus was classified as a keratocyte, whereas a round nucleus indicated an inflammatory cell. The observers who counted and quantified the cells were blinded to the information of which images were the control and which were from the injury group. However, in many cases, because the NM injury was so severe, it was not too difficult to notice which sample was from the control group and which was from the NM injury group.

### 2.4. Immunohistochemistry for COX-2 and FN1

IHC was performed to visualize the expression of COX-2 and FN1 in response to mechlorethamine gel injury. Paraffin-embedded tissue slides were deparaffinized in xylene and rehydrated. Antigen retrieval was conducted in 1x citrate buffer (Cell Signaling Technology, Cat No. 14746, USA) at 98°C for 30 seconds, followed by 10 minutes at 90°C using a pressure cooker. Slides were incubated with freshly prepared 3% hydrogen peroxide for 10 minutes, then blocked with Tris-buffered saline with Tween® 20 (TBST) containing 5% normal goat serum and 2.5% bovine serum albumin at room temperature for 1 hour. After blocking, slides were incubated with primary antibodies (rabbit anti-COX2 (1:300, Cell Signaling Technology, Cat No. 12282, USA) and rabbit anti-FN1 (1:100, Cell Signaling Technology, Cat No. 26836, USA)) diluted in SignalStain® Antibody Diluent (Cell Signaling Technology, USA) overnight at 4°C. On the following day, slides were washed with TBST and incubated with HRP-conjugated secondary antibody (SignalStain® Boost, HRP, Rabbit, Cell Signaling Technology, Cat No. 8114, USA) for 30 minutes at room temperature. Chromogenic detection was performed using the Epredia™ DAB Quanto Detection System (Fisher Scientific, Cat No. TA125QHDX, USA) for 3 minutes. Tissue sections were imaged under a microscope, capturing 3−7 sequential 10x images for quantification using Olympus cellSens Dimension software. COX-2 or FN1-positive cells were quantified and averaged per sample as the percentage of positive area per total tissue area or as the number of positive objects (including cells and extracellular matrix components) per mm^2^ of epithelial and stromal area, respectively.

### 2.5. Statistical analysis

One-way analysis of variance (ANOVA) with multiple comparisons (Kruskal-Wallis post hoc test) or two-tailed unpaired Student’s t-test or Mann-Whitney test was performed accordingly to identify statistical differences between the controls and test groups, using GraphPad Prism (version 10.1.2). Data is representative of at least 3 independent experiments with 2 or more samples per group. Power analysis was performed with a confidence of 0.8, and alpha 0.05. p < 0.05 was considered significant.

## 3. Results

### 3.1. Mechlorethamine gel induced cornea injuries macroscopically

We first developed an NM-induced cornea injury model using *ex vivo* porcine corneas and 0.016% mechlorethamine gel. Fig 1A shows a diagram of the lateral view of the unwounded organ culture of the porcine cornea setup. The top view of a healthy, clear cornea is seen in Fig 1B. Following the application of mechlorethamine to the cornea, an opaque area was evident at the place where NM was applied (Fig 1C, D). The injuries are similar macroscopically to what has been well reported in the literature [32]. The injuries induced in our model, however, have a much more recognizable injury edge/boundary (Fig 1C, D). The less diffused injury than most other mustard injury models (either SM or NM injury) suggests that mechlorethamine gel has the advantage of localized and high concentration release of NM, which is an advantage for experimental and safe use. The gas form of SM or the liquid form of NM, when applied to the cornea, may have less control of localized concentration, thus less control of injury severity, and permeating the air and solution poses significant safety concerns. Less control of localized concentration may result in variation of cellular and tissue damage in experiments.

### 3.2. Mechlorethamine gel induced histopathology, similar to that of SM- or NM-injuries

**Fig 2:**
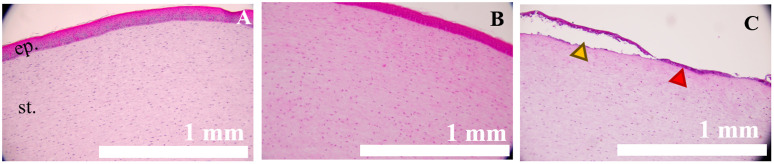
Corneal exposure to mechlorethamine gel for 5 min induces de-epithelialization, typically seen in nitrogen mustard (NM)-induced corneal injury. A. Corneal model with healthy-looking epithelium and stroma. B. Cornea incubated with filter paper (FP) disk, alone, for 5 min. The cornea shows a healthy epithelium and stroma at 0 h post-exposure. C. Cornea exposed to mechlorethamine gel for 5 min, showing complete epithelial-stroma separation (yellow arrowhead) and thinning (red arrowhead) at 0 h post-exposure. H&E staining. N = 3-5. Magnification 10x. Scale bars = 1 mm. ep. = epithelium; st. = stroma.

We then determined histopathological changes following the treatment of mechlorethamine gel. Corneas were fixed immediately after wounding (denominated 0 h hereafter) and H&E staining was performed on healthy, unwounded corneas (control), corneas with filter paper only (FP control), and corneas exposed to mechlorethamine gel for 5 or 15 min (NM 5 min and NM 15 min respectively) to observe the epithelial histopathology post-injury. Healthy epithelium was seen in unwounded and FP control corneas (Fig 2A, B, respectively); whereas epithelial-stroma separation (yellow arrowhead) and epithelial thinning (red arrowhead) were observed in corneas exposed to mechlorethamine gel for 5 min (Fig 2C).

**Fig 3:**
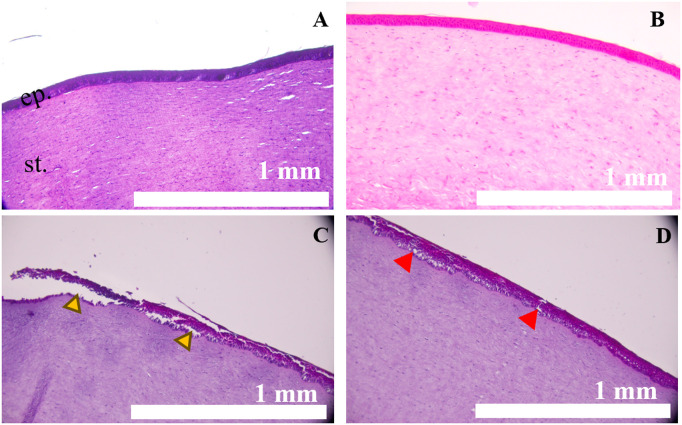
Corneal exposure to mechlorethamine gel for 15 min induces de-epithelialization and epithelium-stroma separation, typically seen in nitrogen mustard (NM)-induced corneal injury. A. Corneal model with healthy-looking epithelium and stroma. B. Cornea incubated with FP disk alone, for 15 min. The cornea showed a normal epithelium and stroma. C. Cornea exposed to mechlorethamine gel for 15 min, showing epithelial loss (yellow arrowheads). D. Cornea exposed to mechlorethamine gel for 15 min, showing epithelial-stroma separation (red arrowheads) at 0 h post-exposure. H&E staining. Magnification 10x. N = 3-4. Scale bars = 1 mm. ep. = epithelium; st. = stroma.

Additionally, unwounded and FP control corneas exhibited healthy epithelium (Fig 3A, B, respectively) when incubated for 15 min. On the other hand, “complete” epithelial-stroma separation (yellow arrowheads) and “partial” epithelium-stroma separation (red arrowheads) in corneas exposed to mechlorethamine gel for 15 minutes are shown in Fig 3C.

**Fig 4:**
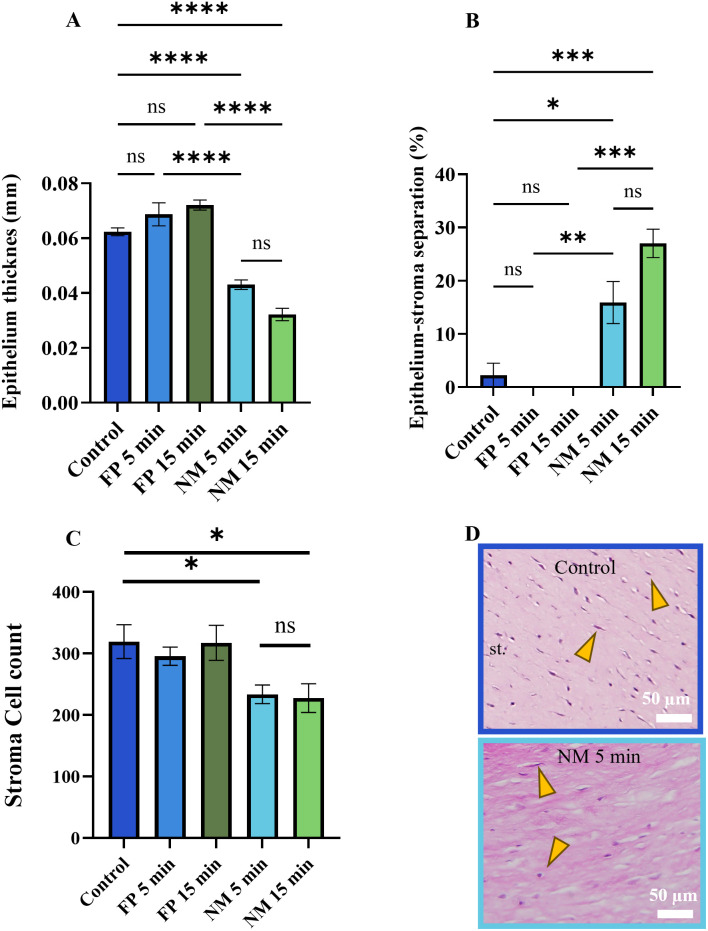
Mechlorethamine gel causes epithelium thinning, epithelium-stroma separation, and decreased total stroma cell count. A) Epithelium thickness decreased, and B) the percentage of epithelium-stroma separation increased after NM exposure. C) The total number of cells in the stroma (yellow arrowheads in panel D) decreased after NM exposure. D) Representative images of the stroma in control and NM 5 min. Data presented as Mean ± SEM. ANOVA with Kruskal-Wallis test and Student’s t-test. *p < 0.05, **p < 0.01, ***p < 0.001, **** p < 0.0001. ns = no significance. N = 3-5. Scale bars = 50 µm. st. = stroma.

We quantified epithelium thickness, epithelium-stroma separation, and total stroma cell counts (6,10,14,29,30) and found that average epithelium thickness was significantly reduced in both NM 5 and 15 min, from healthy control 0.062 ± 0.011 mm to 0.043 ± 0.017 mm and 0.032 ± 0.016 mm, respectively (Fig 4A). Further analysis showed that epithelium-stroma separation (Fig 4B) was significantly higher in corneas exposed for 5 and 15 min to NM (15.8%, p = 0.03; and 27%, p = 0.0003, respectively) compared to healthy controls (2.25%).

Total stroma cell count decreased significantly after NM exposure (average 233 ± 45.9, p = 0.01; and 227 ± 57.5, p = 0.03 for 5 and 15 min, respectively), in comparison to healthy control (average 319 ± 61.5 cells). There was no significant difference in total cell count in the stroma between samples exposed to NM for 5 and 15 min (p = 0.8, Fig 4C).

### 3.3. Mechlorethamine gel induced inflammatory and fibrosis responses

**Fig 5:**
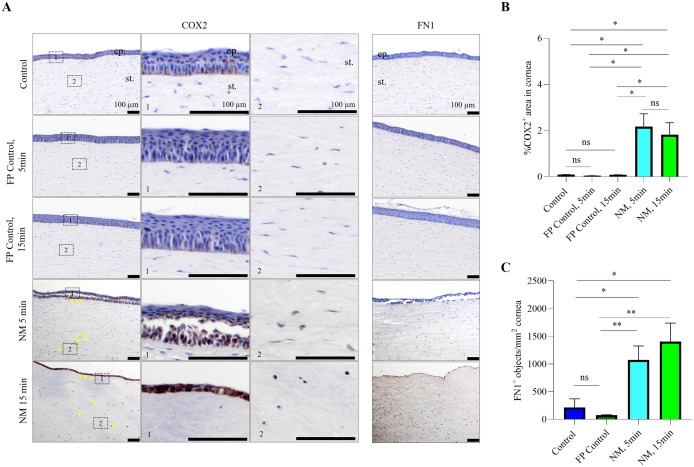
Mechlorethamine gel-induced production of COX-2 and FN1 (inflammatory and fibrotic markers, respectively) is typically seen in NM corneal injury models. Porcine corneas were injured with mechlorethamine gel for 5 and 15 min, and then immediately fixed. IHC showed expression of COX-2 and FN1 in both NM injured corneas. Representative images (A) and quantification (B and C) of immunohistochemistry staining of COX2- or FN1-positive cells in NM-induced corneal injury and controls. High magnification frames on the right of the COX-2 panel show the distribution of COX-2 positive cells in the epithelium and stroma of the NM-treated groups. Yellow arrows point to the COX2-positive keratinocytes in disrupted epithelium and stroma. Data is representative of at least 3 independent experiments, with 2 ~ 4 samples in each group were qualified using unpaired t-test or Mann-Whitney test for statistics. *p < 0.05, **p < 0.01, ns = no significant. Scale bars = 100µm. ep. = epithelium; st. = stroma.

To determine inflammatory and fibrosis responses, we stained for the inflammation marker COX-2 and fibrosis marker FN1 after mechlorethamine gel injury. COX-2 and FN1-positive cells were quantified and averaged per sample as the percentage of positive area per total tissue area or as the number of positive objects (including cells and extracellular matrix components) per mm^2^ of epithelial and stromal area, respectively. Immunohistochemistry showed that both COX-2 and FN1 were highly expressed in mechlorethamine gel-injured corneas, for both 5- and 15-min exposure times (Fig 5A).

We quantified positive labeling of COX-2 and FN1 in the corneas as the % of positive area over total tissue area (COX-2) or the number of positive objects in a defined area (FN1). In the corneas treated with mechlorethamine gel for either 5 min or 15 min, positive labeling of COX-2 and FN1 is statistically significantly higher than that in non-treatment control and FP-controls (Fig 5B).

## 4. Discussion

Here, we describe a safer, novel, and easy NM-induced corneal injury model, where we used mechlorethamine gel to induce an NM injury. This epithelial histopathology is characteristic of NM corneal injuries [10,13,14,18,33]. We observed histopathology typical to that reported in well-established mustard models [6,9,10,13,18,20,34–37]. We expect such a model will significantly facilitate research to understand the mechanisms of SM- and NM- induced ocular injuries, as well as to develop therapeutics.

SM is an alkylating agent and represents a significant threat to the population due to its ease of fabrication, stockpiling, and deployment [1]. Even minimum exposure to SM causes devastating injuries to the eye [1]. NM also has significant limitations, since it remains a very hazardous material that requires specific protective equipment. Those limitations prevent most research laboratories from studying mustard injuries. Consequently, models to study mustard injuries are not available to regular ocular research laboratories, hindering the development of therapeutics.

This mechlorethamine gel does not pose health and safety risks for researchers. The onset of the injury was within minutes after exposure to the gel (Fig 1C). We found that mechlorethamine gel exposure (for both 5 and 15 min) causes epithelium thinning, epithelium stroma separation, changes in corneal cellularity, and increased expression of inflammatory and fibrosis markers. These histopathological features are typical in well-established SM- and NM- models [9,10,12–14,18,19,33,36–39]. Furthermore, our model is consistent with the key lesions in MS keratopathy, e.g., loosening of the epithelium [7], keratocyte loss, and irregularities in epithelium thickness [40].

Mechlorethamine gel induced the expression of COX-2 and FN1 immediately after exposure. COX-2 has been reported as a critical mediator in NM and SM-induced inflammation in the cornea [6,10,13,14,19,33,37]. FN1, a key molecule related to scar formation in the cornea, is expressed during the early phases of wound healing [41,42]. Joseph et al. showed an altered expression of FN1 in rabbit corneas after SM injury [34]. Research involving *in vivo* ocular experiments established structural alterations, inflammation, neovascularization, and opacity when eyes were exposed to SM for less than 4 minutes [32,43–45]. We, therefore, suggest that COX-2 and FN1 production in porcine cornea following NM exposure is the same or very similar to that following SM exposure. Observations of COX-2 and FN1 expression were at time points of at least 24 h in other models. By using our unique model and protocol, these markers could be detected much earlier. Future evaluation should include the detection, at 24 h following injury, of COX-2 and FN1 using this model for better comparison.

There are limitations regarding the quantification of cells in the stroma. The total number of cells decreased following NM exposure, suggesting that some cells in the stroma underwent apoptosis, as shown before [4]. However, others have reported an increased number of cells in the stroma. The main component of this higher number comes from inflammatory cell infiltration, which is likely to be the case *in vivo* models [14]. Additionally, Alemi et al. (2023) reported a higher number of activated keratocytes by day 3, peaking at day 7 *in vivo* [46]. We proceeded to quantify the cellularity in the stroma following the methodology reported by others [6,10,14,19,34] below the injury area. A flat nucleus was classified as a keratocyte, whereas a round nucleus indicated an inflammatory cell [6]. What looked like keratocyte cell counts decreased significantly after NM exposure in comparison to the controls. On the other hand, the number of “inflammatory cells” increased significantly after NM injury (see Figure in S1 Fig), suggesting an inflammatory response. Further tests need to include immunostaining methods to confirm the identity of keratocytes and immune cells, for example, by the use of CD68 and CD4 markers for macrophages and monocytes [47,48], and subsequently quantify positive cells for each type.

Most of the studies describe histopathology after 24 h of mustard exposure. For the safety of the researchers, initial evaluations were done 24 h post-injury to minimize or avoid the risk of SM vapor emission [49]. Charkoftaki et al. assessed corneal structure post NM injury but found no obvious morphological changes 3 h after liquid NM exposure [20]. In their experiments, liquid NM was tested at 1 or 10 mg/mL, and exposure times of 5, 10, and 15 min. At 3 h following exposure, rabbit corneas showed no obvious changes in epithelium and stroma. The authors pointed out that increasing concentration and exposure time to NM caused severe damage to the central cornea. No obvious changes in epithelium and stroma at 3 h are likely due to the relatively low concentration of NM in the tissue. Our results showed structural changes in the porcine cornea soon (in minutes) after exposure, suggesting that the mechlorethamine gel has a localized higher concentration of NM, thus detectable focal injuries shortly after exposure. This is the advantage of the localized release of NM of the mechlorethamine gel to ensure less diffusion, thus ensuring the safety of using the gel in experiments, the same as safe use for patients at home.

Mustard injury is initiated with a chemical reaction of alkylating, which happens immediately when the injury agents reach biomolecules, proteins, DNA, and lipids. Plenty of studies have demonstrated that injury can occur within hours following sulfur mustard exposure in the eye, skin, and respiratory systems. Some older models did not see the injury (pathological changes), most likely due to the dose and duration of exposure, and the time and indices examined [50–52]. Altogether, these findings suggest that there is an early injury soon after NM exposure, similar to that reported in the eyes, skin, and respiratory system due to the nature of chemical reactions upon mustard agents, alkylating proteins, and DNA. Such chemical reactions are expected to happen within minutes of mustard exposure. The initial injuries are likely to be followed by secondary pathological changes of cells and tissue responses to the chemical insults (e.g., apoptosis, immune response, fibrosis), particularly pronounced in the *in vivo* models than *in vitro* due to subsequent inflammation. One advantage of using the safe NM corneal injury model presented herein is that researchers can evaluate safely the effects of NM exposure immediately after injury, facilitating the identification of early or possibly new molecules and mechanisms.

In conclusion, we report a novel, safer, and practical model of NM-induced corneal injury. The epithelial histopathology and expression of inflammation and fibrotic markers, shown in this mechlorethamine gel model, are consistent with those in well-established SM- and NM-induced corneal injury models, thus providing a practical and safe model that can be used in any laboratory to study vesicant-induced injuries in the cornea, and for the development of novel therapeutics.

## Supporting information

S1 Table Reagents used to prepare culture medium for ex vivo corneal culture. (PDF)

S1 Fig Mechlorethamine gel may cause decreased keratocyte and inflammatory cell count. (PDF)
