# Supplementary material for: A practical and safer model of nitrogen mustard injury in cornea
Source: PLoS One. 2025 Jul 3;20(7):e0327622. doi: 10.1371/journal.pone.0327622 (PMC12225829; doi:10.1371/journal.pone.0327622)
Supplement: S1 Table — (PDF) [file pone.0327622.s001.pdf]

S1 Table. Reagents used to prepare culture medium for ex vivo corneal culture.

Reagent	Final concentration	Cat No.	Manufacturer	Country
DMEM/F-12	NA	11330032	Life Technologies	USA
RPMI 1640 vitamins solution	1% v/v	R7256	Sigma-Aldrich	Germany
ITS liquid media supplement	1% v/v	I3146	Sigma-Aldrich	Germany
L-Glutathione reduced	1 µg/mL	G6013	Sigma-Aldrich	Germany
L-Glutamine	1%	25030081	Thermo Fisher Scientific	UK
MEM non-essential amino acids solution	1%	11140050	Thermo Fisher Scientific	USA
Sodium Pyruvate	1 mM	11360070	Thermo Fisher Scientific	USA
Antibiotic Antimycotic solution (ABAM)	1%	A5955	Sigma-Aldrich	Germany
Gentamicin	50 µg/mL	G1397	Sigma-Aldrich	Germany
2-O-s-D-Glucopyranosyl-L-ascorbic acid	1 mM	SMB00390	Sigma-Aldrich	Germany
