# Supplementary material for: A practical and safer model of nitrogen mustard injury in cornea
Source: PLoS One. 2025 Jul 3;20(7):e0327622. doi: 10.1371/journal.pone.0327622 (PMC12225829; doi:10.1371/journal.pone.0327622)
Supplement: S1 Fig — (PDF) [file pone.0327622.s002.pdf]

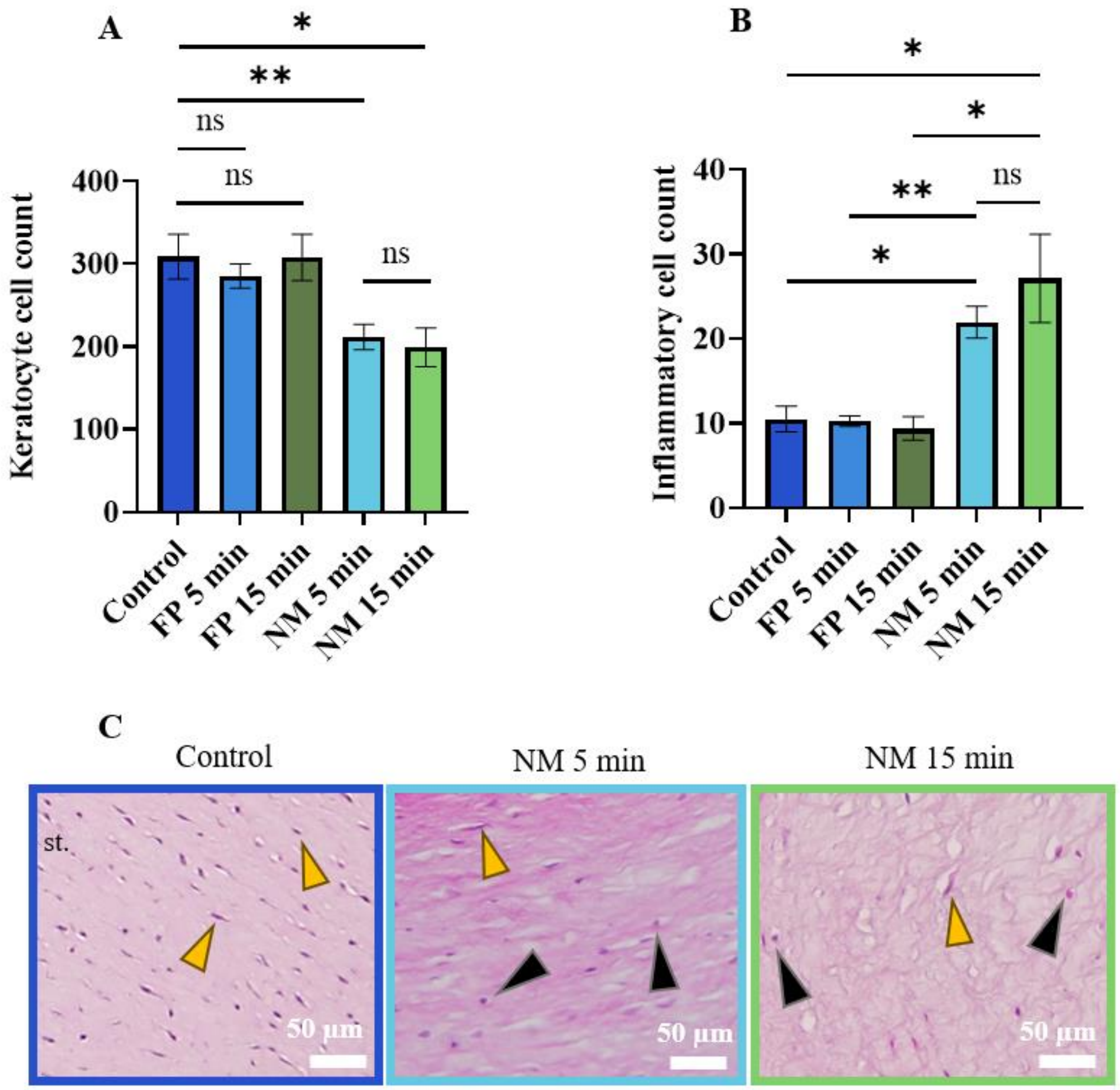

S1 Figure. Mechlorethamine gel may cause decreased keratocyte and inflammatory cell count. A) Keratocyte cell count decreased (yellow arrows in panel C) after NM exposure whereas B) the inflammatory cells (black arrowhead in panel C) count increased. Data presented as Mean ± SEM. ANOVA with Kruskal-Wallis test and Student's t-test. *p <0.05, **p< 0.01,***p< 0.001, **** p<0.0001. ns= no significance. N=3-5. Scale bars = 50 µm. st.= stroma.
